# Intensity-adjustable pain management with prolonged duration based on phase-transitional nanoparticles-assisted ultrasound imaging-guided nerve blockade

**DOI:** 10.1186/s12951-022-01707-z

**Published:** 2022-11-24

**Authors:** Bin Qiao, Xinye Song, Weiyi Zhang, Ming Xu, Bowen Zhuang, Wei Li, Huanling Guo, Wenxin Wu, Guangliang Huang, Minru Zhang, Xiaoyan Xie, Nan Zhang, Yong Luan, Chunyang Zhang

**Affiliations:** 1 Department of Medical Ultrasonics, The First Affiliated Hospital of Sun Yat-Sen University, Guangzhou, 510080 People’s Republic of China; 2 Department of Anesthesiology, The First Affiliated Hospital of Dalian Medical University, Dalian, 116011 People’s Republic of China

**Keywords:** Controlled release, Ultrasound, Theranostics, Cell membranes, Analgesia duration

## Abstract

**Background:**

The lack of a satisfactory strategy for postoperative pain management significantly impairs the quality of life for many patients. However, existing nanoplatforms cannot provide a longer duration of nerve blockage with intensity-adjustable characteristics under imaging guidance for clinical applications.

**Results:**

To overcome this challenge, we proposed a biocompatible nanoplatform that enables high-definition ultrasound imaging-guided, intensity-adjustable, and long-lasting analgesia in a postoperative pain management model in awake mice. The nanoplatform was constructed by incorporating perfluoropentane and levobupivacaine with red blood cell membranes decorated liposomes. The fabricated nanoplatform can achieve gas-producing and can finely escape from immune surveillance in vivo to maximize the anesthetic effect. The analgesia effect was assessed from both motor reactions and pain-related histological markers. The findings demonstrated that the duration of intensity-adjustable analgesia in our platform is more than 20 times longer than free levobupivacaine injection with pain relief for around 3 days straight. Moreover, the pain relief was strengthened by repeatable ultrasound irradiation to effectively manage postoperative pain in an intensity-adjustable manner. No apparent systemic and local tissue injury was detected under different treatments.

**Conclusion:**

Our results suggest that nanoplatform can provide an effective strategy for ultrasound imaging-guided intensity-adjustable pain management with prolonged analgesia duration and show considerable transformation prospects.

**Supplementary Information:**

The online version contains supplementary material available at 10.1186/s12951-022-01707-z.

## Introduction

Postoperative pain management continues to be a significant clinical and scientific challenge with acute discomfort in patients [1]. However, for specific individuals among 10% of surgical patients, acute postoperative pain lasts longer than the standard period for tissue repair and progresses to chronic pain [2, 3]. The use of opioids to relieve acute and chronic pain has increased over the past decade, associated with an increase in prescription opioid misuse, abuse, and overdose-related death [4–6]. Despite considerable efforts that have been paid to develop effective pain management strategies with local anesthetic medicines, their analgesia duration is still limited [7, 8]. As a result, for many decades, there has been engaged in developing sustained release methods that would deliver a prolonged period of local anesthetic [9, 10]. However, such a method still lacks a way to modify drug release profiles and cannot respond to changing patient demands. Therefore, it is necessary, urgent, but challenging to develop an efficient strategy to achieve intensity-adjustable pain management with prolonged duration and finally improve local anesthetic [11].

Research into improving the pain management outcomes after surgery focused on the drug-delivery systems responsive to external stimuli [11–14]. Responsive drug delivery systems could achieve intensity-adjustable pain management with the reduced application of opioids in the clinic. Moreover, the controllable drug delivery system can tail analgesia duration, location, and intensity with repeated external stimuli. Because of its variable wavelength, irradiance area, and exposure time, light has been exploited as a potential external trigger [12, 15, 16]. However, because of its low tissue penetration depth, the light-triggered local anesthetic release strategy suffered from a problem endemic to light in medicine [17–19]. Ultrasound with high tissue penetration seemed to be an attractive tool for guiding analgesia because it effectively visualizes nerve, skeletal tissues, and surrounding vascular structures [20–23]. Compared to blind nerve blocks relying on doctors’ experience, an ultrasound-guided nerve block can improve the success rate of clinical analgesia and reduce injury-related complications [24]. Therefore, attempts to achieve the underlying ultrasound imaging-guided intensity-adjustable pain management are highly required.

New ultrasound imaging and pain management strategies have been significantly developed in parallel [25–27]. Clinical application of ultrasound has demonstrated its biological safety and provided evidence in enhancing the delivery efficiency of anti-tumor drugs [28–30]. Because of the inert cavitation effect and the increase in interstitial fluid flow, ultrasonic microbubbles might promote medication delivery [31, 32]. However, microbubbles are unstable under physiological circumstances, and the short half-life of a single dosage prevents a sustained analgesic impact. Compared to microbubbles, the liquid-to-gas phase-transitional nanomedicine stands out as an optimal partner for ultrasound imaging because of the relatively high stability profiles in theranostics. Ultrasound irradiation may trigger the phase-transition process and transform liquid-phase perfluoropentane (PFP) into a gas phase for ultrasound imaging [33, 34]. More importantly, the phase-transitional ultrasonic contrast agent could change the acoustic environment and facilitate drug delivery based on the inert cavitation effect [34, 35]. Even so, pain management is still limited by the insufficient retention time of the nanoparticles in vivo.

Drug delivery systems prepared with nanotechnology have shown promise in diverse disease diagnosis and treatment. Among these systems, liposomes made up of phospholipid bilayers were anticipated to be explored as potential options for prolonging the duration of pain management [36, 37]. Unfortunately, the previous research mainly focuses on employing local analgesics to alleviate pain, while the potential of liposomes in intensity-adjustable pain management is still in its infancy [38]. Moreover, the retention time of these liposome-based nanoparticles still leaves much to be desired [39, 40]. To date, the decoration with red blood cell membranes (RBCM) has been effectively utilized as a “don’t eat me” signal to enhance the accumulation of diverse nanomedicine in the tumor sites [41, 42]. The mechanism of RBCM for prolonging the retention time in vivo can be attributed to the CD47, a self-recognition protein on the surface of RBCM, which could prevent macrophage activation and uptake in vivo [43]. Therefore, we assumed RBCM decorated nanomedicine could escape from the immune system for an extended half-time, which is anticipated to be beneficial for sustained analgesia.

Herein, we described an experimental postoperative pain management model system that links the controlled release process of local anesthetics and its subsequent biological response to manage pain. Specifically, we employed the liquid-to-gas phase-transitional technology to reduce the ultrasonic intensity. Compared to the nanoparticles without cavitation effect, it has the potential to reduce the energy intensity of ultrasonic irradiation with comparable cavitation effect and avoid additional side effects. Recent evidence has emphasized the prominent role of using external stimuli to manage pain intensity, leading us to focus on ultrasound-triggered intensity-adjustable pain management. Based on the above analysis, we developed an RBCM-decorated liposome loaded with phase-transitional perfluoropentane (PFP) and a small molecular local anesthetic levobupivacaine, denoted as RBCM-lipsomes@levobupivacine-PFP (RLBP). In the nanoplatform, RLBP could initiate the liquid-to-gas phenomenon and produce a gas environment. The changed acoustic environment could facilitate the ultrasound-triggered release of levobupivacaine with moderate-intensity ultrasound and provide an imaging signal for contrast-enhanced ultrasound imaging. Meanwhile, RLBP exhibited prolonged retention time after being decorated with RBCM to achieve prolonged pain management in vivo. The ultrasound irradiation synergizes with RLBP and could result in adjustable pain intensity by ultrasound-triggered levobupivacaine release under ultrasound imaging-guided. Our results indicate that RLBP plus ultrasound irradiation significantly alleviates local pain with adjustable intensity properties. Here, we emphasize the promise of a strategy for intensity-adjustable pain management with prolonged duration under ultrasound imaging-guided.

## Materials and methods

### Materials

Levobupivacaine hydrochloride (Bupi) was obtained from Aladdin (Shanghai). Distearoyl phosphatidyl ethanolamine-polyethylene glycol 2000 (DSPE-PEG2000), dipalmitoyl phosphatidylcholine (DPPC), and cholesterol were purchased from AVT (Shanghai) Pharmaceutical Tech Co. Ltd. 4,6-diamidino-2-phenylindole (DAPI) and BCA protein detection kit was obtained from Beyotime Biotechnology (Shanghai). Agarose was purchased from Invitrogen. Endothelial cell medium was purchased from Sciencell Corporation. A standard cell counting kit-8 assay (CCK-8) was purchased from Dojindo Biotechnologies (Japan). PFP was obtained from J&K Science Ltd. Dialysis bags (cutoff 3500 Da) were purchased from Yuanye Biotechnology (Shanghai). All of the chemical reagents were analytical or reagent grade without further purification.

### Preparation of lipsomes@levobupivacine-PFP (LBP)

LBP was prepared by a facile one-step filming-rehydration method. Briefly, 2 mg DSPE-PEG2000, 5 mg DPPC, and 1.5 mg cholesterol were dissolved in trichloromethane (5 mL) in a flask with round-bottomed. A homogeneous layer film (liposomes) was formed after 1 h of spinning under vacuum evaporation at 50 °C. Subsequently, levobupivacaine hydrochloride (4 mg) in distilled water (4 mL) and 400 μL PFP were added under intermittent sonication (100 W, 3 s on, and 3 s off) for 2 min in an ice bath. Finally, the LBP nanoparticles were fabricated after repeated centrifugation (8000 rpm, 10 min) and stored at 4 °C for further use.

### Extraction of RBC membranes

Differential centrifugation was used to acquire RBC membranes. Briefly, blood was collected from BALB/c mice and centrifuged (3000*g*, 5 min, 4 °C). The RBCs were washed twice with 1× PBS and then suspended in 0.25× PBS to make them break in a hypotonic solution. Next, the suspension was centrifuged (16,600*g*, 30 min), and the cytoplasmic components were depleted, resulting in pink sediment. The obtained RBC membranes were stored at − 80 °C before use.

### Preparation of RBCM-lipsomes@levobupivacine-PFP (RLBP)

RLBP was prepared by sonication of the RBC membranes and liposomes film with a weight ratio of 1:1 in an ice bath. Subsequently, levobupivacaine hydrochloride (4 mg) in distilled water (4 mL) and 400 μL PFP were added under intermittent sonication (100 W, 3 s on, and 3 s off) with a Sonifier (Sonics VCX500) for 2 min in an ice bath. Finally, the RLBP nanoparticles were fabricated after repeated centrifugation (8000 rpm, 10 min) and stored at 4 °C for further use.

### Membrane fusion confirmation

The fusion of the liposomes and the RBC membranes was verified based on a previously reported method [44]. Briefly, highly lipophilic fluorescent green dyes 3,3′-dioctadecyloxacarbocyanine perchlorate (DiO) and red dyes 1,1′-Dioctadecyl-3,3,3′,3′-Tetramethylindodicarbocyanine, 4-Chlorobenzenesulfonate Salt (DiD) were used to label the RBC membranes and liposomes, respectively. DiD-labeled liposomes were prepared by the same method as demonstrated above, except that DiD (500 μg, Beyotime, China) was dissolved in the solution. To prepare the DiO-labeled RBC membranes, DiO dissolved in ethanol was added dropwise into the RBC membranes (2 mg/mL) and incubated for 30 min at room temperature. After that, the resulting solution was centrifuged at 16,000×*g* for 30 min to eliminate the free DiO. The DiO-labeled RBC membranes and DiD-labeled liposomes were resuspended in PBS before use. The nanoparticles were then tested using flow cytometry with the receiving wavelength filter of 530 nm and 680 nm.

### Ultrasound-responsive levobupivacaine release in vitro

The RLBP nanoparticles were centrifugated (8000 rpm, 10 min) several times to remove the excess levobupivacaine. Subsequently, the RLBP nanoparticles (5 mg/mL) were set in a dialysis bag (cutoff 3.5 KDa) and immersed in 20 mL of PBS solution. Then, these dialysis bags were put into a constant temperature shaker at 37 °C (JINGHONG, China) and stirred at 160 rpm for 12 h. The dialysis bags were put in an ultrasound field (0.6 W/cm^2^, 1.0 MHz, duty cycle 50%) for 15 min 6 times at selected times. The dialysis bags without ultrasound irradiation were set as controls. Subsequently, the released medium (5 mL) was collected, and the released levobupivacaine was examined by UV–Vis spectrum. The levobupivacaine release rates were determined using the formula: concentration of released levobupivacaine/total concentration of levobupivacaine. The experiment was performed three times.

### Cytotoxicity evaluation of RLBP in vitro

Sciencell Biotechnology Co., Ltd. provided HUVEC cells; the HUVEC cells (10^4^ per well) were seeded in a 96-well plate overnight to adhere. Then the plates were added with different concentrations of RLBP nanoparticles (0.05, 0.1, 0.2, 0.3, 0.4, 0.5, and 0.6 mg/mL) for 4 h. After that, the old cell medium was replaced with a fresh medium. The obtained plates were put in an ultrasound field (0.6 W/cm^2^, 1.0 MHz, duty cycle 50%) for 2 min, and the supernatant was removed. The cells were added with RPMI 1640 medium (90 μL) and CCK-8 assay (10 μL). The cell viability was tested by measuring the absorbance at 450 nm using a microplate reader (Varioskan LUX, USA) after 2 h incubation at 37 °C. The 96-well plate without ultrasound treatment was set as a control. The cytotoxicity of RLBP to HUVEC cells was also tested. Briefly, HUVEC cells (10^4^ cells) were seeded in a 96-well plate and incubated to settle overnight. Subsequently, RLBP nanoparticles with different concentrations (0.05, 0.1, 0.2, 0.4, 0.6, 0.8, and 1.0 mg/mL) were added into the wells. After incubation at 37 °C for 24 h or 48 h, the cell viability was tested with a standard CCK-8 assay, as noted. For laser confocal microscope (CLSM) observation, HUVEC cells (10^5^ cells per well) were planted into glass-bottled cell culture dishes and incubated overnight to allow settling. Subsequently, the medium was discarded, and the dishes were added with RLBP nanoparticles (1.0 mg/mL) in the cell medium. After co-incubation for 4 h, the cells were then irradiated with ultrasound. Then the cells were stained with calcein-AM (2 × 10^6^ μM) and propidium iodide (PI) (2 × 10^6^ μM) at 37 °C for 30 min. The cells were observed with CLSM (LSM710, Zeiss) after three washes with PBS. The experiment was performed three times.

### Ultrasound responsive liquid-to-gas phase transition examination of RLBP nanoparticles

The liquid-to-gas capability of RLBP nanoparticles was evaluated after ultrasound irradiation with the same intensity (0.6 W/cm^2^). First, 1 mL of RLBP with a concentration of 1 μg/mL was added into a self-made agarose gel phantom and irradiated for 25 min. Subsequently, the agarose gel phantom was imaged by an ultrasonic imaging device (Visual Sonics Inc., Canada) with CEUS-Mode. The ultrasonic images were collected and analyzed by ultrasonic quantitative analysis software (Chongqing Medical University) to record their sound intensity. Immediately, the RLBP nanoparticles after treatment for different times (0, 5, 10, 15, 20, and 25 min) were collected to observe under light microscopy (Nikon, DS-Ri2). The liquid-to-gas phase-transitional performance of the RLBP nanoparticles was then evaluated after ultrasound irradiation with different ultrasound intensities (0.2, 0.6, 1.0, 1.6 W/cm^2^) for 8 min. Briefly, 1 mL of RLBP with a concentration of 1 μg/mL was added into a self-made agarose gel phantom and irradiated with ultrasound; the agarose gel phantom was imaged by an ultrasonic imaging device with CEUS-Mode. The experiment was performed three times.

### Animals

Adult male Balb/c mice (4–6 weeks) were obtained from Guangdong experimental animal center, and the animal care committee approved the experimental protocol of Sun Yat-sen University. The mice were kept in an SPF-grade animal experiment center for 2 weeks before subsequent behavioral evaluation.

### Procedures for building the model of incision pain in mice

The mice with incision pain were built according to the techniques demonstrated in a previous study [45]. Briefly, 2% isoflurane was used to sedate the mice before surgery. Subsequently, the operation area was cleaned with a 10% povidone-iodine solution. After building a 2 mm longitudinal incision at the proximal edge of the heel, the local muscle was sliced and lifted. Last, 4-0 nylon thread was used to suture the local skin, followed by antibiotic ointment to prevent incision infection.

### Local RLBP administration around the isolated nerve

The sciatic nerve was injected locally around the isolated nerve site with levobupivacaine hydrochloride, RLBP suspension, LBP suspension, RLB suspension, or RLP suspension with 0.05 mg levobupivacaine hydrochloride (100 μL, n = 5 mice per group). Then the hind leg was exposed to ultrasound with the same intensity after the immobilization of mice in specific groups.

### CEUS/NIRF imaging with RLBP nanoparticles in vivo

To evaluate the phase transitional phenomenon of RLBP, the mice injected with RLBP Nanoparticles (100 μL) were exposed to ultrasound irradiation at different time points. Then the mice were imaged with the ultrasonic imaging device (Aplio i900) with an 18 MHz probe. The obtained figures were analyzed with ultrasonic quantitative analysis software (Chongqing Medical University) to record their sound intensity. For NIRF Imaging, the mice were injected with RLBP and then imaged with a Xenogen IVIS Spectrum device (PerkinElmer, USA). Similarly, the images were analyzed with the software provided by the PerkinElmer company.

### Measurement of mechanical hyperalgesia in vivo

The behavioral performance was tested on mice to evaluate mechanical hyperalgesia to non-noxious mechanical stimuli. The mice were arranged to acclimate to the environment before the test for 30 min, where the mice were set onto a silk mesh in a plastic cage. An intelligent needle probe was used to stimulate the back paw of the mice with a steadily increased force. When the mice raised their left paw, the pressure was automatically recorded as the paw mechanical threshold (PWT). The data measured on the day before the experiment’s start are considered baseline values. The test was repeated 5 times in each mouse, and their average was calculated.

### Measurement of thermodynamic hyperalgesia in vivo

The behavioral performance was tested on mice to evaluate thermodynamic hyperalgesia to non-noxious thermal stimuli. The mice were set onto a heating base of 55 °C. The paw withdrawn latency (PWL) was shown on the screen of the Hargreaves device (KW-LB, Kaerwen Inc., China) when the left hind paw was lifted. The test was stopped with a shutoff time of 20 s. Three independent measurements proceeded, and more than 5 min of space–time was set to protect the mice from additional burns. The data measured on the day before the experiment’s start are considered baseline values. The experiment was performed independently five times.

### Hematoxylin–eosin (H&E), toluidine blue and immunofluorescence staining for evaluating biosafety in mice

The sciatic nerve and surrounding tissues were obtained at 3 and 28 days of local administration combined with ultrasound irradiation at the same intensity (0.6 W/cm^2^, 1.0 MHz, 2 min, duty cycle 50%). Moreover, different groups harvested the heart, liver, spleen, lungs, and kidneys from the mice. All the tissues were rinsed with physiological saline and sliced. After being stained with H&E, the slices were photos with a digital automatic biopsy scanner (Kfbio, China). The thoracic cavity of the mice was perfused with PBS and 4% paraformaldehyde via the left ventricle after anesthesia. After different treatments combined with ultrasound at the same intensity, the ipsilateral L5 Dorsal Root Ganglion (DRG) was harvested at 54 h post-surgery. The tissues were precooled and immersed in a 30% sucrose solution for immunofluorescence staining.

### Enzyme-Linked immunosorbent assay (ELISA) for evaluating biosafety in mice

The mice were sacrificed at 3 days and 14 days after establishing the model of incision pain. On the second day, the sciatic nerve was injected locally around the isolated nerve site with levobupivacaine hydrochloride and RLBP suspension with 0.05 mg levobupivacaine hydrochloride (100 μL, n = 3 mice per group). Subsequently, the sciatic nerve and its surrounding muscle tissue were harvested. According to the vendor’s instructions, cytokines (IL-1β, TNFα, and IFNγ) were analyzed with ELISA kits (Jingmei, China). The experiment was performed three times.

### Statistical analysis

The data were presented as mean ± standard derivation. Corresponding data analyses were performed using GraphPad Prism 9 software by Student t-test, one-way ANOVA, and further compared with Turkey’s multiple analysis.

## Results and discussion

### Preparation and characterization of RLBP nanoparticles

**Fig. 1:**
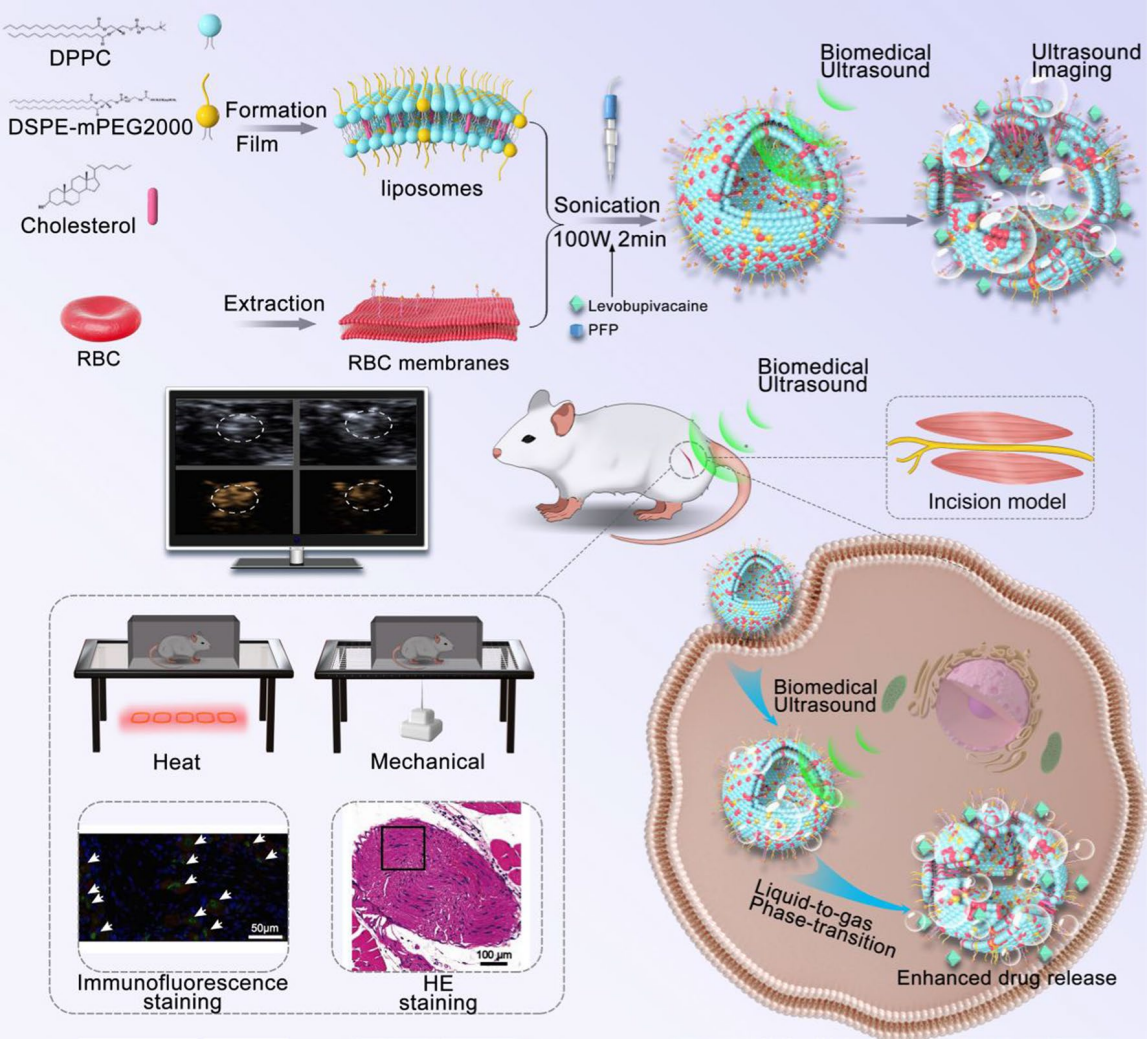
**A** Fabrication of the RLBP nanoparticles. **B** Schematic illustration of intensity-adjustable pain management with prolonged duration for ultrasound imaging-guided nerve blockade in vivo

**Fig. 2:**
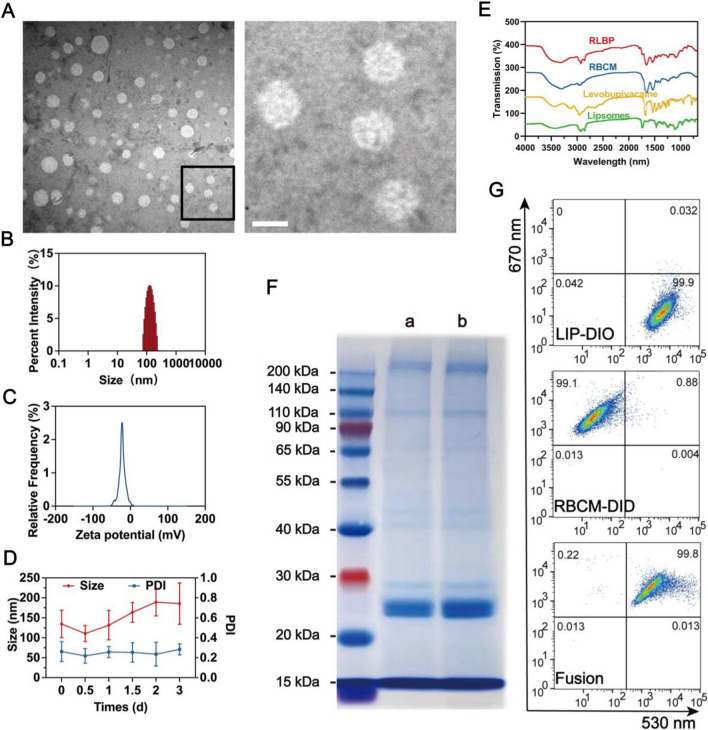
**A** The representative TEM images of the RLBP nanoparticles (left) and corresponding zoom images (right). The scale bars are 500 nm (left) and 100 nm (right). **B** Size distribution of the RLBP nanoparticles. **C** Zeta potential variations in the preparation of RLBP nanoparticles. **D** The stability of the RLBP nanoparticles was monitored with size distribution and PDI. **E** FTIR spectrum of RLBP, RBC membranes, levobupivacaine, and liposomes. **F** SDS-PAGE analysis of the (a) RLBP nanoparticles and the (b) RBC membranes. **G** Flow cytometry detects the fusion effect of DIO-labeled liposome and DID-labeled RBCM in the RLBP nanoparticles

Figure 1 shows the schematic illustration of the RLBP nanoparticles’ production process. The thin film hydration method prepared the LBP nanoparticles according to the reported articles [46]. To obtain the RBC membranes and liposomes hybrid RLBP nanoparticles, RBC membranes were fabricated using the typical hypotonic strategy. The RBCs were isolated from the whole blood of Balb/c mice, and then the cells were ruptured in the hypotonic medium. The RBC membranes were obtained by centrifugation to remove the intracellular contents, and the obtained products were preserved at – 80 °C before use. PFP with a liquid-to-gas phase-transitional characteristic was selected as the critical component for ultrasound imaging. Moreover, the loading of PFP can modulate the stability of the nanoparticles, which could particularly benefit the drug delivery process under ultrasound irradiation (Additional file 1: Figure S1). The prepared RLBP nanoparticles showed a spherical shape in the representative transmission electron microscopy (TEM) images (Fig. 2A). In addition, levobupivacaine hydrochloride was loaded into liposomes to facilitate pain management capability. The average size of RLBP is 134.5 ± 34.3 nm, and the zeta potential of − 23.7 ± 0.8 mV is measured by dynamic light scattering (DLS) (Fig. 2B and C). The pure RBC membrane found was difficult to form stable nanoparticles without adding liposomes, but the nanoparticles showed ideal stability at 4 °C (Fig. 2D). The UV–Vis absorption spectra of RLBP nanoparticles retained the characteristic absorption peak of levobupivacaine hydrochloride at 263 nm (Additional file 1: Figure S2). The drug loading capacity of levobupivacaine hydrochloride was determined to be 73.9 ± 6.7% based on the established standard curve (Additional file 1: Figure S3). Moreover, the Fourier-transform infrared (FTIR) spectrum of the RLBP nanoparticles showed characteristic absorption peaks of levobupivacaine at approximately 2656 cm^−1^, further confirming the successful loading of levobupivacaine molecules (Fig. 2E). Moreover, the FTIR spectrum of the RLBP nanoparticles retains the absorption peaks of RBC membranes at 1656 cm^−1^ and 1542 cm^−1^, as well as the absorption peaks of liposomes at 2922 cm^−1^ and 2853 cm^−1^. The above FTIR results suggest that the RLBP nanoparticles consisted of RBC membranes, liposomes, and levobupivacaine. In addition, the SDS-PAGE result showed that the proteins of RBC membranes were almost retained in the RLBP nanoparticles (Fig. 2F). To verify the successful fusion of the RBC membranes and the liposomes, membrane fusion studies were performed. The RBC membranes were labeled with DID, and the liposomes were labeled with DIO dyes. The liposomes and RBC membranes overlay in the RLBP nanoparticles could be verified using flow cytometry. The result showed merged fluorescent images in the RLBP nanoparticles, indicating both the liposomes and RBC membranes were fused in the nanoparticles (Fig. 2G). Above all, these results showed the successful fabrication of RLBP nanoparticles.

### Ultrasound responsive contrast-enhanced ultrasound (CEUS) imaging and levobupivacaine hydrochloride release in vitro

**Fig. 3:**
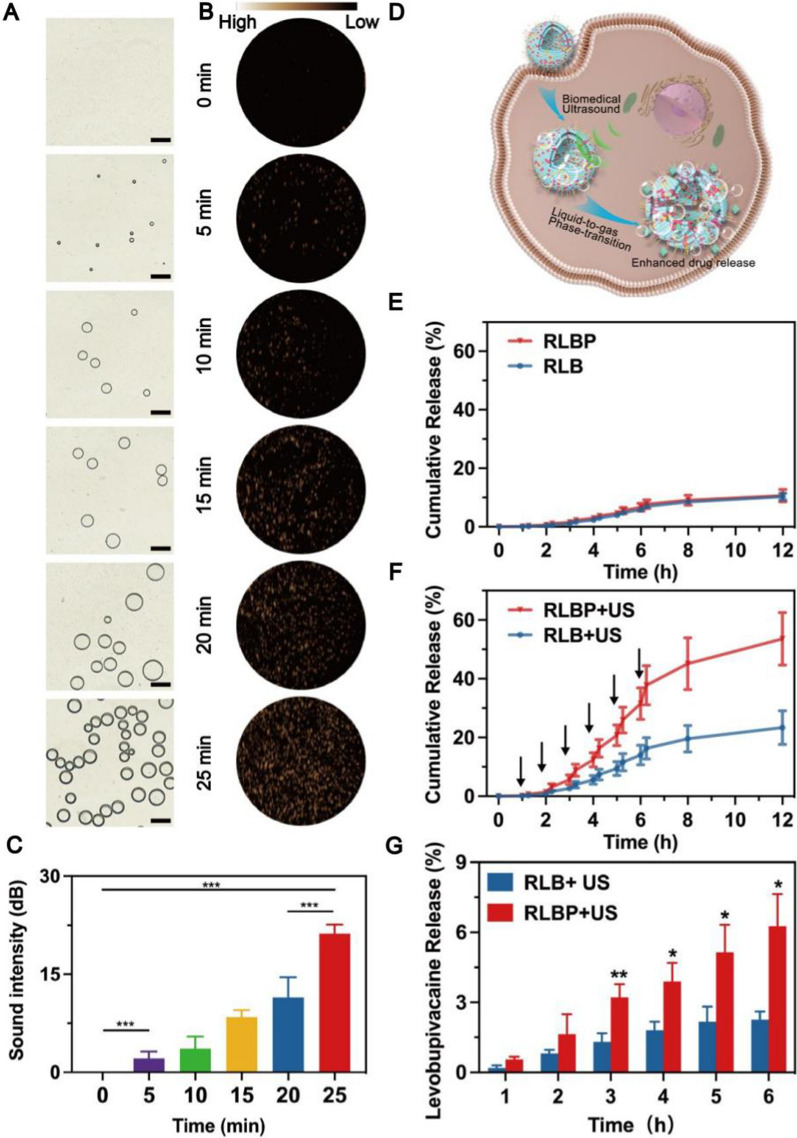
**A** Optical microscope images of the RLBP nanoparticles after ultrasound irradiation. The scale bar is 25 μm. **B** CEUS images of the RLBP nanoparticles after ultrasound irradiation at different times. **C** Quantitative analysis of the sound intensity in the CEUS images. **D** Schematic illustration of the phase-transitional process of the RLBP nanoparticles with ultrasound irradiation and ultrasound-enhanced levobupivacaine release profile. **E** Cumulative release of levobupivacaine without ultrasound irradiation. **F** Cumulative release of levobupivacaine with ultrasound irradiation. **G** Quantitative levobupivacaine release after each cycle of ultrasound irradiation (n = 5, *p < 0.05, **p < 0.01, compared by Students’ t-test)

Ultrasound was applied in pain management due to its outstanding performance in guiding the nerve blockade in the clinic. However, the short half-life of traditional microbubbles cannot afford long-term imaging guidance because traditional microbubbles are unstable in physiological settings. Liquid-to-gas phase-transitional ultrasound agent stands out as an ideal partner for ultrasound imaging compared to standard ultrasonic microbubbles, which hold the advantage of comparatively high stability in theranostics. Therefore, to confirm the ultrasonic imaging characteristic of RLBP, optical microscope and CEUS images of RLBP under different ultrasonic treatments in agarose gel were recorded. The size of RLBP nanoparticles increased from the nanometer scale to the micron scale with the prolonged ultrasonic irradiation duration (Fig. 3A), which indicated that the RLBP nanoparticles could experience a liquid-to-gas process, conferring on its capability for CEUS. The maximum microbubble volume captured on the optical microscope was around 25 μm, which showed advantageous for drug release due to the change in Laplace pressure, which satisfies the requirements of in our investigation. Subsequently, the CEUS images of RLBP nanoparticles were recorded, and the sound intensity was analyzed (Fig. 3B, C). The results showed a significant increase in the imaging signal after 5 min of ultrasound irradiation. The increases in CEUS signal can be attributed to the formation of microbubbles. Moreover, increased ultrasonic irradiation intensity can result in a more pronounced phase-change impact and increased CEUS imaging signal. Altogether, these findings verified the RLBP nanoparticles’ remarkable ultrasound-responsive imaging characteristics, allowing them to be used as a CEUS imaging agent for pain management. The gas generation after the phase-transitional process could result in an improved ultrasound-responsive drug release due to the ultrasonic cavitation effect mechanism (Fig. 3D) [47]. Therefore, we tested the in vitro ultrasound-triggered levobupivacaine hydrochloride release behavior. Few levobupivacaine hydrochlorides released from the RLBP nanoparticles were observed throughout the 12 h experimental period without ultrasound irradiation (Fig. 3E). In addition, levobupivacaine hydrochloride release from the RLB nanoparticles without PFP loading under ultrasound irradiation was much lower than that from the RLBP nanoparticles (Fig. 3F). The difference in the responsiveness between the RLB and RLBP nanoparticles could be attributed to the liquid-to-gas phenomenon in the presence of PFP, suggesting the improved cavitation effect could enhance the levobupivacaine release. Moreover, single ultrasound irradiation released more levobupivacaine at each ultrasound treatment cycle (Fig. 3G), which can be attributed to the gradually enhanced cavitation effect after repeated ultrasound irradiation. These features made RLBP essential for CEUS-guided intensity-adjustable pain management.

### In vitro biosafety of RLBP examination combined with ultrasound irradiation

**Fig. 4:**
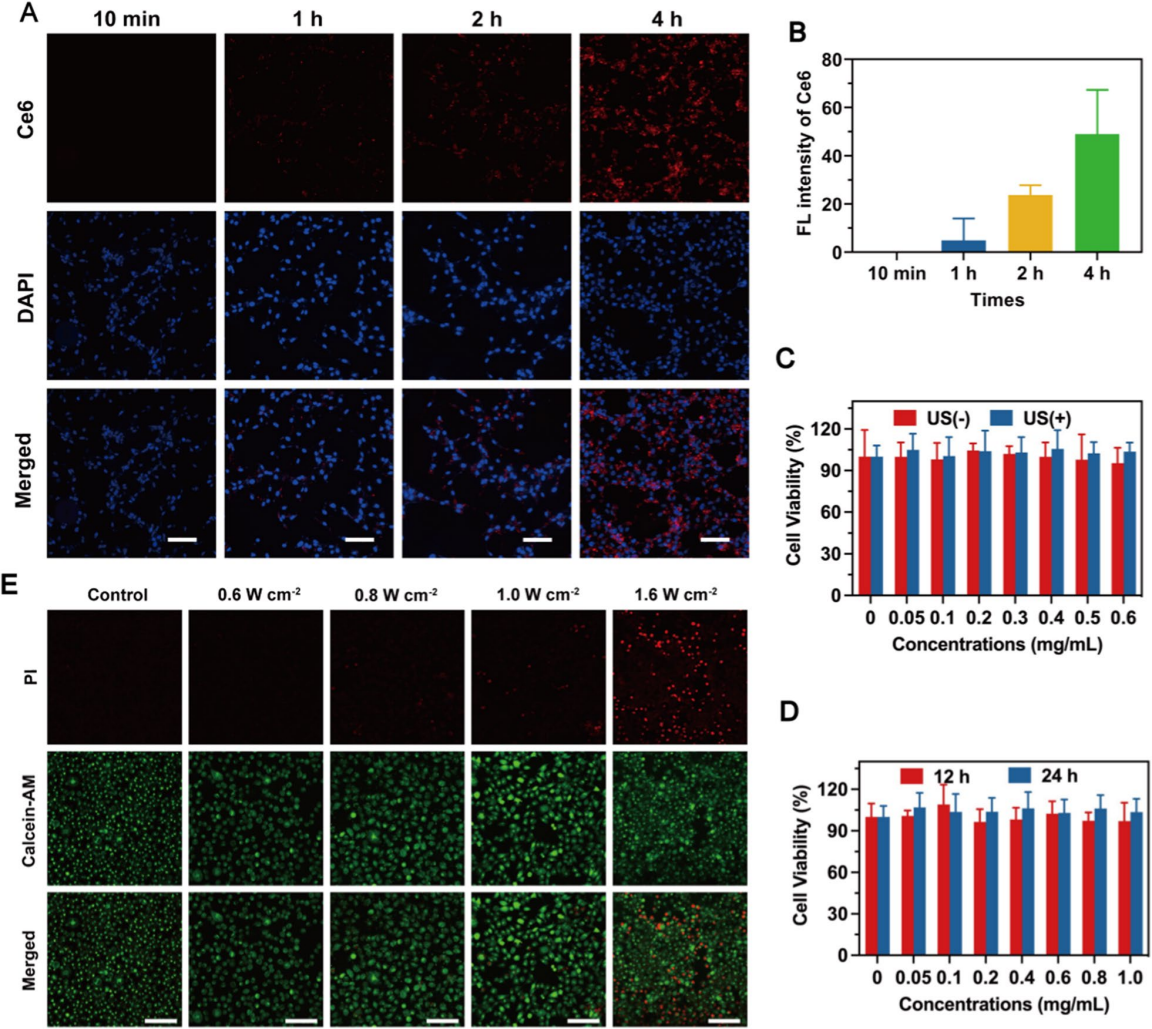
**A** Cell internalization of HUVEC cells treated with Ce6 doped RLBP nanoparticles and **B** corresponding quantitative data. The scale bars are 50 μm. **C** Cell viability assay of the RLBP nanoparticles incubated with HUVEC cells with or without ultrasound irradiation. **D** Cell viability assay of RLBP nanoparticles-treated HUVEC cells for different times. **E** Calcein-AM and PI co-staining images of DRG cells treated by RLBP nanoparticles with different ultrasound irradiation intensities

Endothelial cells are distinct cell lines from normal tissue. Therefore, we choose the HUVEC cell line to detect cell internalization and apoptosis. The fluorescence intensity of RLBP increased with the extension of incubation duration (10 min–4 h). These findings further validate the effectiveness of cellular internalization of RLBP for further applications (Fig. 4A and B). The cells were incubated with the RLBP nanoparticles for 4 h and then exposed to ultrasound irradiation with different intensities for 2 min. The live/dead cell staining result showed that the higher power (0.8 W/cm^2^, 2 min) can induce DRG cell death after the addition of RLBP nanoparticles (0.6 mg/mL) for 4 h in our study (Fig. 4E). Therefore, the ultrasound intensity used in our study is 0.6 W/cm^2^. Given the potential use of RLBP nanoparticles as contrast agents for CEUS-guided pain management, the biosafety of RLBP in vitro was tested with a standard CCK-8 assay at increasing concentrations from 0.05 to 0.6 mg/mL. The cells were incubated with the RLBP nanoparticles for 4 h and then exposed to ultrasound irradiation (0.6 W/cm^2^, 1.0 MHz, 50% duty cycle) for 2 min. The result showed no significant reduction in cell viability in the ultrasound irradiation group, suggesting the pain management process is highly compatible (Fig. 4C). Moreover, the cells co-incubated with RLBP for 12 h and 24 h showed similar cell viability, further indicating the high biosafety of the RLBP nanoparticles in vitro (Fig. 4D). We also performed live/dead cell staining to detect the cell apoptosis in stained HUVEC cells, and no prominent cells were stained with red fluorescent apoptotic cells after ultrasound irradiation (Additional file 1: Figure S4). In addition, the ROS levels after incubation with Ce6-doped RLBP were detected using 2′,-7′-dichlorofluorescein diacetate (DCFH-DA), a ROS indicator that is converted to fluorescent 2′,-7′-dichlorofluorescein (DCF), which emits green fluorescence under CLSM observation. After ultrasound irradiation (0.6 W/cm^2^, 1.0 MHz, 50% duty cycle), RLBP nanoparticles showed negligible green fluorescence (Additional file 1: Figure S5), indicating minimal ROS generation. Above all, these results demonstrated the high biocompatibility of RLBP for in vivo pain management.

### Pain management monitored by CEUS/NIRF imaging in vivo

**Fig. 5:**
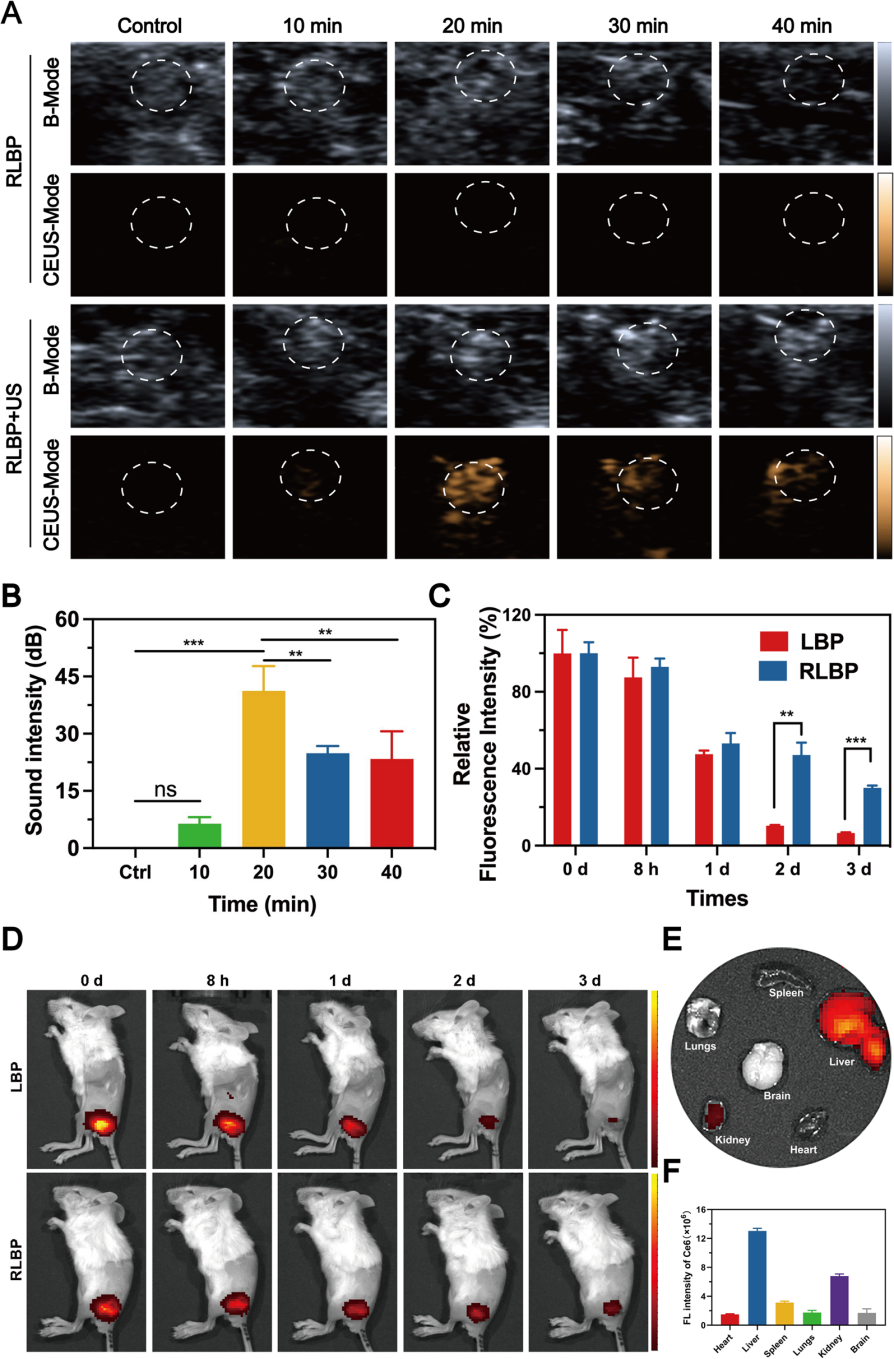
**A** In vivo B-Mode and CEUS-Mode ultrasonic images around the sciatic nerve after injection with RLBP nanoparticles. The circle marks the treated sciatic nerve. **B** Quantitative analysis of sound intensity in the CEUS-Mode ultrasonic images. **C** Quantitative analysis of relative fluorescence signal of **D** NIRF images of the mice treated by the RLBP nanoparticles and the LBP nanoparticles. (n = 3, *p < 0.05, **p < 0.01 compared by Students’ t-test) **E** NIRF images of the major organs (including heart, liver, spleen, lungs, kidney, and brain) in mice after injection of RLBP and **F** corresponding quantitative data

Currently, ultrasound imaging has been regarded as an essential means for the visualization of nerve tissue in the clinic. However, monitoring the levobupivacaine distribution remains a complex problem to solve [11, 48, 49]. Although microbubbles could provide a methodology to monitor the levobupivacaine distribution, the low stability of microbubbles makes it difficult to provide long-term pain management. Compared to traditional microbubbles, the RLBP could be used as a multifunctional probe for relatively long-term pain management with imaging guidance due to its relatively high stability. After ultrasound irradiation at different times, the RLBP + US group showed an enhanced CEUS signal around the sciatic nerve in mice, especially with 20 min of ultrasound irradiation (Fig. 5A and B). However, the RLBP without the ultrasound group showed no detectable CEUS signal due to the lack of external stimuli. Accordingly, the RLBP could be regarded as an ultrasound-responsive nanoplatform for CEUS-guided pain management. Subsequently, Ce6 has been carried out to label the RLBP nanoparticles for analyzing the retention time of different nanoparticles in vivo. In vivo semiquantitative analyses of the fluorescence signal of the RLBP nanoparticles compared to that of the injection time revealed more than fourfold elevations compared to the LBP nanoparticles (without RBC membranes decoration) since 2 days, which indicated the advantages of RLBP nanoparticles as an agent for analgesia with prolonged duration (Fig. 5C and D). The long period can be attributed to the presence of RBC membranes due to the expression of CD47, which is essential for in vivo pain management. In addition, levobupivacaine hydrochloride was metabolized by hepatic cytochrome P450, according to previously reported articles [50]. The RLBP nanoparticles last more than 3 days, and the RLBP was mainly distributed in the liver at 24 h after local injection of the RLBP according to the NIRF images (Fig. 5E and F). Moreover, no significant NIRF signal was observed in the brain, indicating minimal nanoparticles overcome the blood–brain barrier.

### In vivo intensity-adjustable pain management with RLBP nanoparticles

**Fig. 6:**
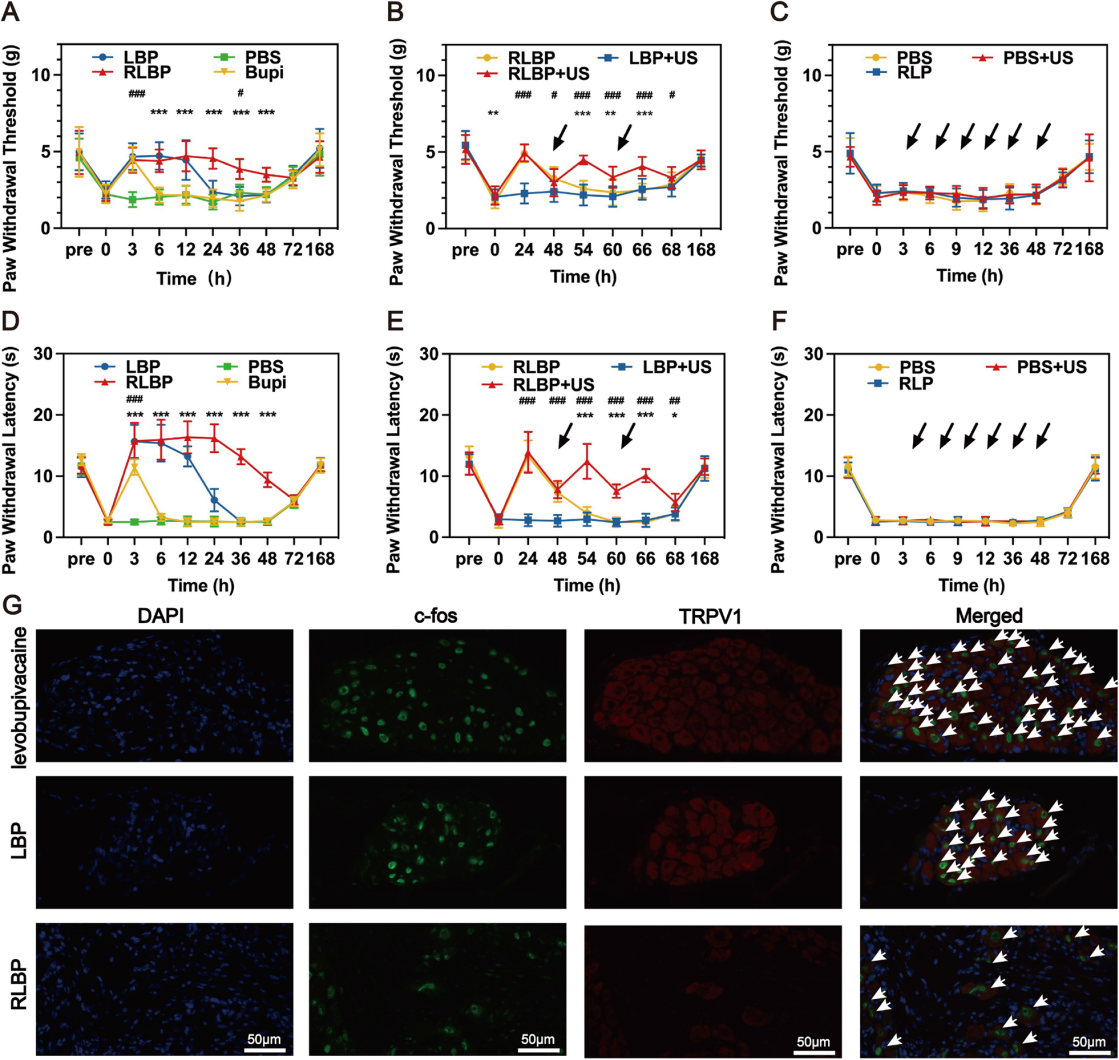
**A** Mechanical threshold before and after different treatments (The data were shown as mean ± SD, n = 5, ^#^p < 0.05, ^###^p < 0.001 was compared with Turkey’s multiple analysis between PBS and levobupivacaine, ***p < 0.001 was compared between RLBP and levobupivacaine). **B** Mechanical threshold before and after different treatments. The black arrows in the figure mean ultrasound irradiation. (The data were shown as mean ± SD, n = 5, ^#^p < 0.05, ^###^p < 0.001 was compared with Turkey’s multiple analysis between LBP + US and RLBP + US, *p < 0.05, **p < 0.01, ***p < 0.001 was compared with Turkey’s multiple analysis between RLBP and RLBP + US). **C** Mechanical threshold of mice treated by RLP, PBS, and PBS + US. The black arrows in the figure mean ultrasound irradiation. **D** Thermal latency before and after different treatments (The data were shown as mean ± SD, n = 5, ^#^p < 0.05, ^###^p < 0.001 was compared with Turkey’s multiple analysis between PBS and levobupivacaine, ***p < 0.001 was compared with Turkey’s multiple analysis between the RLBP and the levobupivacaine group). **E** Thermal latency before and after different treatments. The black arrows in the figure mean ultrasound irradiation. (The data compared with Turkey’s multiple analysis were shown as mean ± SD, n = 5, ^##^p < 0.01, ^###^p < 0.001 between LBP + US and RLBP + US, *p < 0.05, ***p < 0.001 was compared with Turkey’s multiple analysis between RLBP and RLBP + US). **F** Thermal latency of mice treated by the RLP, PBS, and PBS + US. The black arrows in the figure mean ultrasound irradiation. **G** Immunofluorescence assay of ipsilateral L5 DRG at 54 h after treatment with the RLBP + US, LBP + US, and levobupivacaine + US. The scale bars are 50 μm

An incision pain model evaluated the intensity-adjustable pain management process with the RLBP nanoparticles and their action duration [51]. The mechanical withdrawal threshold and the thermal latency in mice were tested in an incision pain model. The model was successfully verified at 3 h after surgery due to the significantly reduced withdrawal mechanical threshold and thermal latency. Subsequently, the mice were given levobupivacaine, LBP nanoparticles, or RLBP nanoparticles with a dose of 0.05 mg levobupivacaine (100 μL) around the sciatic nerve. The mice injected with 100 μL of PBS were set as a control. Mechanical hyperalgesia and thermal latency were tested to observe the behavioral change. The intensity of the mechanical threshold was recorded using an electrical device with a pinpoint to display the matching pressure. The group treated with levobupivacaine showed a moderate pain block of 3 h, while the PBS group showed no significant pain block after treatment (Fig. 6A). In contrast, the infusion of LBP nanoparticles showed prolonged pain management with a nerve block duration of 12 h, which can be attributed to the effect of nanotechnology in extending the period of local anesthetics [52]. Although several liposome-based nanoparticles have been used to prolong the duration of pain management, the retention time of these nanoparticles still leaves much to be desired [53]. The fabrication of RLBP showed a prolonged analgesic duration of more than 48 h, indicating the decoration with RBC membranes could improve pain management efficacy.

Although several methods have been used to prolong the duration of pain management, the nanoplatforms stills could not respond to external stimuli. Therefore, these nanoplatforms cannot reply to the changing patient demands. A local anesthetic release platform that patients could easily adjust could be beneficial. Thus, the mice were treated with RLBP nanoparticles plus ultrasound to evaluate the intensity-adjustable pain management process. As shown in Fig. 6B, the pain measurement results showed that the mechanical threshold decreased 48 h after surgery, and the ultrasound irradiation could elevate the mechanical threshold, suggesting the successful modulating of the pain with the help of ultrasound. Moreover, the mechanical threshold increased for as long as 6 h, which can be attributed to the unique features of RLBP nanoparticles due to the ultrasound triggering and sustained levobupivacaine release pattern. In the LBP plus ultrasound group, ultrasound was exposed to the mice at 48 h, and no significant elevation on the mechanical threshold was observed, further indicating the decoration of RBC membranes could prolong the duration of pain management.

To eliminate the influence of ultrasound, RBC membranes, and liposomes, the mechanical threshold in the control groups (including the PBS solution group, the PBS solution + US group, and the RLP nanoparticles alone group) was tested. The results showed no significant effect on pain relief among these groups (Fig. 6C), demonstrating that the ultrasound, RBC membranes, and liposomes could not block the incision pain. Notably, the mechanical threshold gradually recovered to the untreated baseline in these groups at 168 h, indicating the formal establishment of the mice model.

The incision pain model was used to assess thermal latency 3 h after surgery. Heat hyperalgesia was evaluated via behavioral tests that produced thermal stimulation at a specified temperature, and the time interval of paw withdrawal was recorded (Fig. 6D–F). Thermal latency lasted less than 3 h in the DBP and PBS groups. In contrast, animals administered LBP through the sciatic nerve increased their thermal latency on 55 °C hot plates to roughly 6 h compared to free levobupivacaine, indicating that LBP may aid pain management (Fig. 6D). Surprisingly, RLBP had a longer analgesic duration than DBP, which might be attributed to the decorating of RBC membranes to achieve a more extended retention period in vivo. Mice injected with RLBP in the US had better thermal latency, indicating that ultrasonic irradiation may improve pain management through the acoustic droplet vaporization effect (Fig. 6E). The PFP encapsulation may provide a gas environment for more robust levobupivacaine release. The PBS, PBS + US, and RDP groups had no significant analgesic effect as controls, indicating that ultrasound-responsive RDBP + US may block incision pain (Fig. 6F).

### In vivo immunofluorescence analysis of L5 dorsal root ganglion (DRG)

Incision pain is accompanied by a decreased nociceptive threshold, increasing the peripheral afferent neurons’ responsiveness [51]. The sciatic nerve is composed primarily of axons from the L4 and L5 DRG; therefore, the ipsilateral L5 DRG was harvested for immunofluorescence analysis. C-fos is an immediate early gene, which could be rapidly reduced upon the activation of the DRG cells [54]. Transient receptor potential cation channel vanilloid subtype 1 (TRPV1) is a nonselective cation channel that could be reduced in tissue injury-related thermal hyperalgesia [55]. Consequently, the neurons were double-labeled with TRPV1 and C-fos to intuitively visualize the pain-related state and confirm the behavioral characteristic in mice. At 54 h post-injection, the labeled with TRPV1 and c-fos reduced obviously in the RLBP group compared to the PBS group and the levobupivacaine group (Fig. 6G), indicating the improved incision pain block with prolonged duration of RLBP + US in our strategy. Above all, these immunofluorescence analysis results further validated the animal behavioral test results.

### In-vivo histocompatibility and neurotoxicity test

**Fig. 7:**
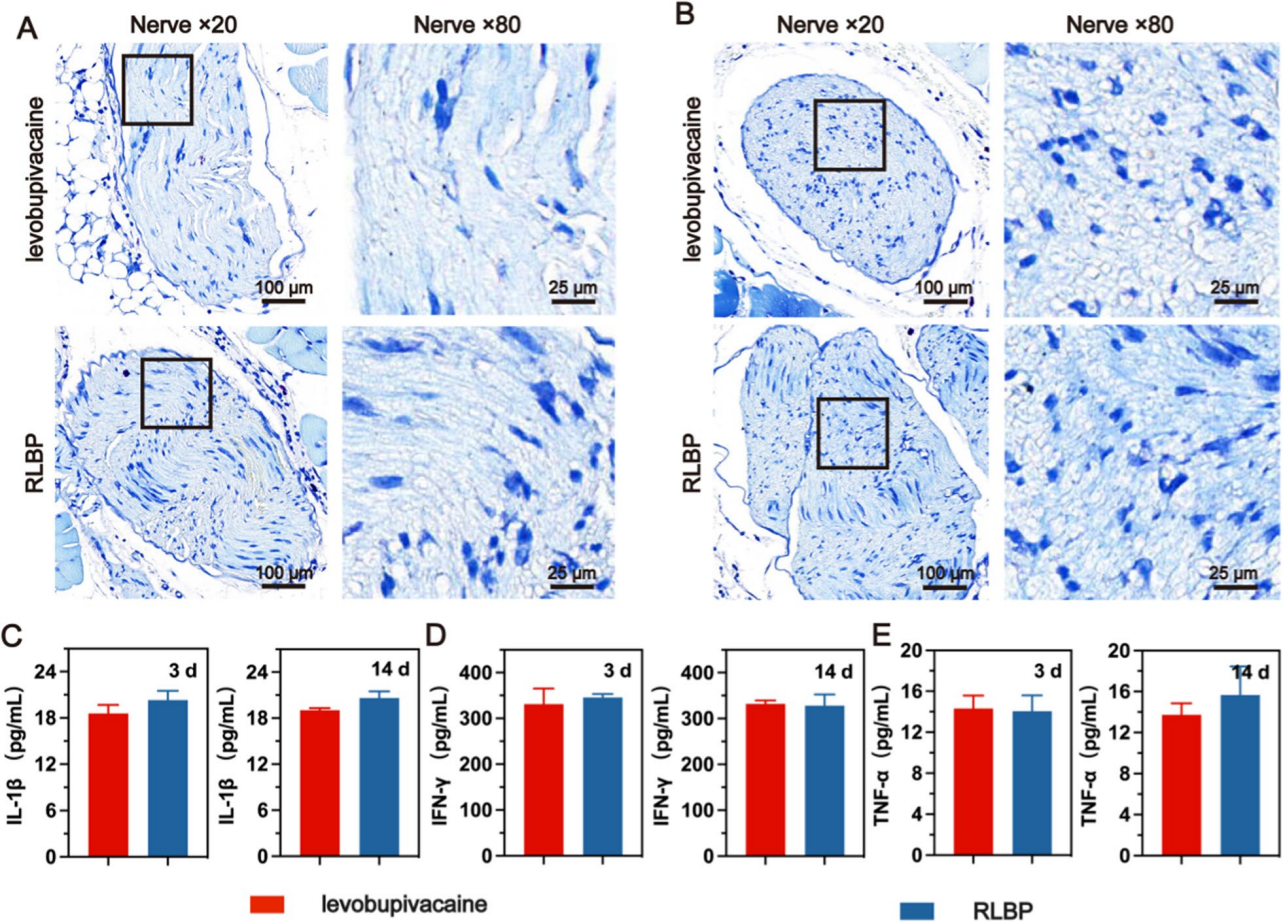
Representative toluidine blue staining images of the sciatic nerve at **A** 3 days and **B** 28 days after levobupivacaine + US and the RLBP + US treatments. **C**–**E** Cytokine levels in tissues after levobupivacaine + US and the RLBP + US treatments at different times

To evaluate the biocompatibility of the therapeutic process in vivo, we evaluate the nerve and muscle tissue sections at 3 days and 28 days after the pain management process by H&E staining (Fig. 7 and Additional file 1: Figure S6). No necrosis and significant inflammatory damage were observed, indicating the superior biosafety of the RLBP nanoparticles in vivo. Because of the low sensitivity of H&E staining in detecting nerve injury, we used toluidine blue staining to stain the nessoids of the nerve cells, which is an important indicator in evaluating the neurotoxicity of nanoparticles. Minimal peripheral nerve damage and decreased axon density at 3 days and 28 days after ultrasound irradiation. This further demonstrated that the RLBP nanoparticles combined with ultrasound irradiation for intensity-adjustable pain management are highly biocompatible.

In vivo biocompatibility of the levobupivacaine and the RLBP nanoparticles was also investigated during the intensity-adjustable pain management process in the major organs (Additional file 1: Figure S7). The H&E staining results treated with levobupivacaine and the RLBP nanoparticles showed no significant tissue damage or inflammatory reaction. Moreover, the cytokines were investigated, and there was no significant increase in the production of TNF-α, IFN-γ, and IL-1β in the RLBP nanoparticles group compared with clinical applicable levobupivacaine (Fig. 7C–E), indicating the strong biosafety of RLBP for in vivo applications.

## Discussion

In this study, we report the development of RBC membranes-decorated nanoparticles for intensity-adjustable pain management with a prolonged duration that might be useful for managing postoperative patient in the clinic. Moreover, our result in the postoperative model in mice raises the possibility that nanoplatform is ultrasound responsiveness to adjust the pain management intensity according to the patient’s demand. Furthermore, these nanoplatforms can prolong the duration of pain management by RBC membrane decoration when analyzed with in vivo models. In addition, we confirmed that the nanoplatform could be guided with ultrasound imaging in the incision pain model.

Although liposome-based nanoparticles have been proven effective over an extended period by continuously releasing high doses of local analgesics, the retention time still leaves much to be desired [13]. The RLBP nanoparticles were produced by the hybrid of extracted RBC membranes and liposomes. They potentially provide an advantage over currently used local anesthetics-loaded liposomes in that their in vivo retention time can be significantly prolonged simply by decorating the nanoparticles with RBC membranes. The proof-of-concept study confirmed that the RBC membranes extracted from ex vivo blood retain the ability to escape from the body’s immune system and the analgesic time was significantly prolonged. As a result, we demonstrated that our strategy could provide a better pain management effect compared to clinically transformable liposomes.

The research on improving the pain management outcomes after surgery that external stimuli could adjust is necessary for patient-centric pain management [12, 13]. Although several efforts have been focused on changing the intensity of pain alleviation with external light, the limited tissue penetration depth hindered the clinical applications. As ultrasound is commonly used in the clinic and therapeutic ultrasound devices are commercially available, intensity-adjustable pain relief with ultrasound was proposed. However, the low stability of traditional microbubbles inhibits a long-term analgesic effect. Compared to conventional ultrasonic microbubbles, the liquid-to-gas phase-transitional ultrasound agent showed relatively high stability; the high stability guarantees the nanoparticles did not undergo a significant phase-transition process in vivo and cannot release levobupivacaine in a burst manner without ultrasound irradiation, which is important for the intensity-adjustable and long duration analgesia.

Another critical issue in the pain management process is ultrasound imaging guidance. Currently, an ultrasound-guided nerve block is widely used in the clinic and has shown excellent success in reducing injury-related complications. The ultrasound-guided pain management can identify the injection site in situ, confirm the injection site, and provide information for triggering drug release. Therefore, it is quite possible to envision that nanoagents could provide ultrasonic imaging signals for guiding nerve block, followed by confirming nerve block and then adjusting the intensity of pain management. Considering that ultrasound irradiation could start the phase transition process and turn liquid-phase PFP into gas-phase so that ultrasound imaging could be done, the producing gas environment could also facilitate drug release based on the inertial cavitation effect. Notably, inertial cavitation could significantly avoid ROS production generated by sonodynamic therapy [56]. In addition, the inertial cavitation effect in the gas environment can dramatically reduce the power of ultrasound stimuli and stimuli-related subsidiary injury [57]. These data support that the cavitation effect enhanced mechanical effect may be more suitable for triggering levobupivacaine release during pain management.

Several studies have indicated that encapsulating several of the most regularly used local anesthetics in liposomes extends the duration of their anesthetic action. Therefore, distinct liposomal formulations encapsulating local anesthetics of the intermediate period (prilocaine, lidocaine, or mepivacaine) improved the duration and intensity of the anesthetic effect compared to plain solutions with these local anesthetics [38, 58, 59]. However, these liposomes’ retention time significantly impedes the duration of regional analgesia. RBC membranes have been used to prolong the retention time of multiple agents [60]. The mechanism of RBCM for extending the retention time in vivo can be attributed to the CD47, a self-recognition protein on the surface of RBCM, which could prevent macrophage activation and uptake in vivo. However, as to our best known, the effect of RBC membranes on extending the duration of their anesthetic action has rarely been reported. We sought to improve the analgesic performance of the RLBP nanoparticles by increasing the retention time. The stranded nanoparticles could enhance the duration of the RLBP nanoparticles and ultrasound responsiveness in vivo. Our research strategy can provide new ideas for developing clinically-convertible local analgesics with the help of nanotechnology.

## Conclusion

In conclusion, for the first time, we report the concept of a strategy for intensity-adjustable pain management with the assistance of the constructed RLBP nanoparticles, which have successfully achieved ultrasound imaging-guided nerve block. The intriguing pain management outcome was based on the unique characteristic of liquid-to-gas phase-transitional nanoparticles. The loaded PFP was converted to a gaseous state to affect the ultrasonic imaging signal to achieve imaging guiding and monitoring. Meanwhile, the formed gaseous PFP can induce a burst release of levobupivacaine after ultrasound irradiation due to the cavitation mechanism to achieve intensity-adjustable pain management. Primarily, RBC membranes were employed to prolong the liposome-based nanoparticles’ analgesic duration, inducing an ultrasound imaging-guided long-lasting pain management. Therefore, this unprecedented “intensity-adjustable pain management with prolonged duration for ultrasound imaging-guided nerve blockade” concept successfully combines the biomimetic nanomedicine with theranostic technology for solving the important issue of clinical pain management, paving a new way for developing efficient pain management strategy with simultaneous long duration and well ultrasound responsiveness.

## Supplementary Information

**Additional file 1: Figure S1.** Optical microscope photographs of RLBP and RBC membrane-constructed nanoparticles. The magnification of the picture is 40×. **Figure S2.** The UV–Vis spectrum of RBCM, LIP-PFP, levobupivacaine, and RLBP. **Figure S3.** The absorbance UV–Vis spectrum of the levobupivacaine in our study. **Figure S4.** Calcein-AM and PI co-staining images of the RLBP nanoparticles with or without ultrasound irradiation. **Figure S5.** Generation of ROS by DCFH staining of DRG cells after being treated with RLBP and RLBP + US. The scale bars are 50 μm. **Figure S6.** Representative H&E images of sciatic nerve and muscle at (A) 3 days and (B) 28 days after levobupivacaine + US and the RLBP + US treatments. **Figure S7.** H&E staining sections of the major organs from the mice at (A) 3 days and (B) 28 days after treatment by different pain management strategies. The scale bar is 100 μm.

## Data Availability

All data generated or analyzed during this study are included in this published article.
