# Supplementary material for: Intensity-adjustable pain management with prolonged duration based on phase-transitional nanoparticles-assisted ultrasound imaging-guided nerve blockade
Source: J Nanobiotechnology. 2022 Nov 24;20:498. doi: 10.1186/s12951-022-01707-z (PMC9694595; doi:10.1186/s12951-022-01707-z)
Supplement: Supplementary file 1 — Additional file 1: Figure S1. Optical microscope photographs of RLBP and RBC membrane-constructed nanoparticles. The magnification of the picture is 40×. Figure S2. The UV–Vis spectrum of RBCM, LIP-PFP, levobupivacaine, and RLBP. Figure S3. The absorbance UV–Vis spectrum of the levobupivacaine in our study. Figure S4. Calcein-AM and PI co-staining images of the RLBP nanoparticles with or without ultrasound irradiation. Figure S5. Generation of ROS by DCFH staining of DRG cells after being treated with RLBP and RLBP + US. The scale bars are 50 μm. Figure S6. Representative H&E images of sciatic nerve and muscle at (A) 3 days and (B) 28 days after levobupivacaine + US and the RLBP + US treatments. Figure S7. H&E staining sections of the major organs from the mice at (A) 3 days and (B) 28 days after treatment by different pain management strategies. The scale bar is 100 μm. [file 12951_2022_1707_MOESM1_ESM.docx]

**Intensity-Adjustable Pain Management with Prolonged Duration Based on Phase-Transitional Nanoparticles-Assisted Ultrasound Imaging-Guided Nerve Blockade**

Bin Qiao^1#^, Xinye Song^2#^, Weiyi Zhang^2^, Ming Xu^1^, Bowen Zhuang^1^, Wei Li^1^, Huanling Guo^1^, Wenxin Wu^1^, Guangliang Huang^1^, Minru Zhang^1^, Xiaoyan Xie^1^, Nan Zhang^1*^, Yong Luan^2*^, Chunyang Zhang^1*^

1. Department of Medical Ultrasonics, The First Affiliated Hospital of Sun Yat-sen University, Guangzhou, 510080, P. R. China;

2. Department of Anesthesiology, The First Affiliated Hospital of Dalian Medical University, Liaoning, 116011, P. R. China;

Correspondence: Chunyang Zhang*, Yong Luan*, Nan Zhang*

E-mail: zhangchy36@mail.sysu.edu.cn, luanyong@dmu.edu.cn, zhangn257@mail.sysu.edu.cn


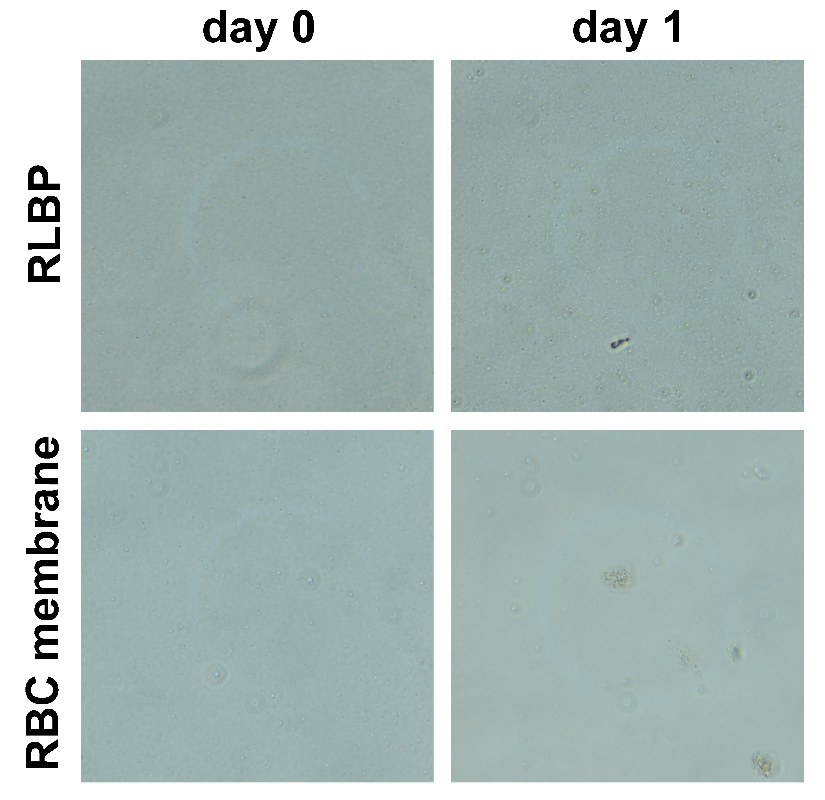


Figure S1. Optical microscope photographs of RLBP and RBC membrane-constructed nanoparticles. The magnification of the picture is 40X.


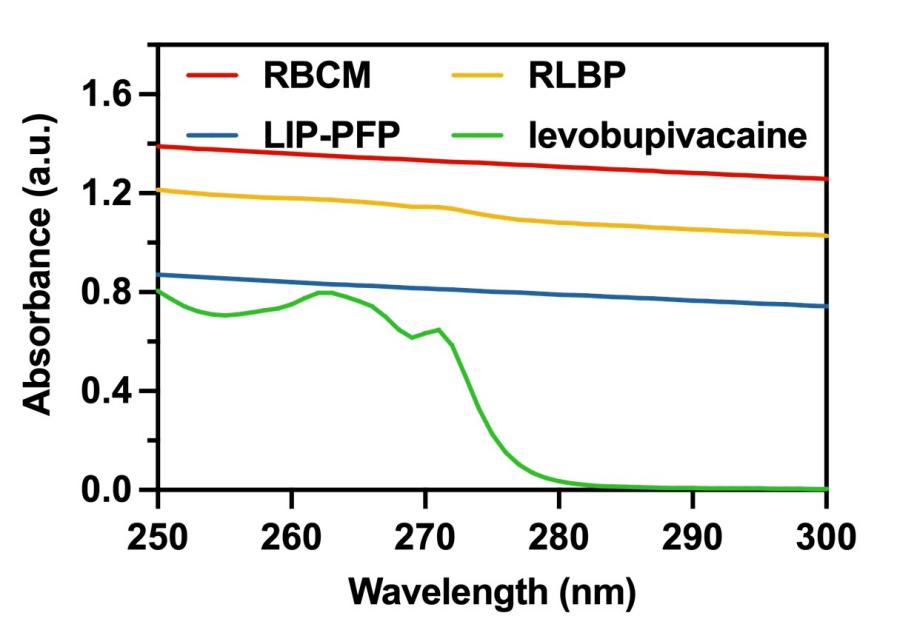


Figure S2. The UV-Vis spectrum of RBCM, LIP-PFP, levobupivacaine, and RLBP.


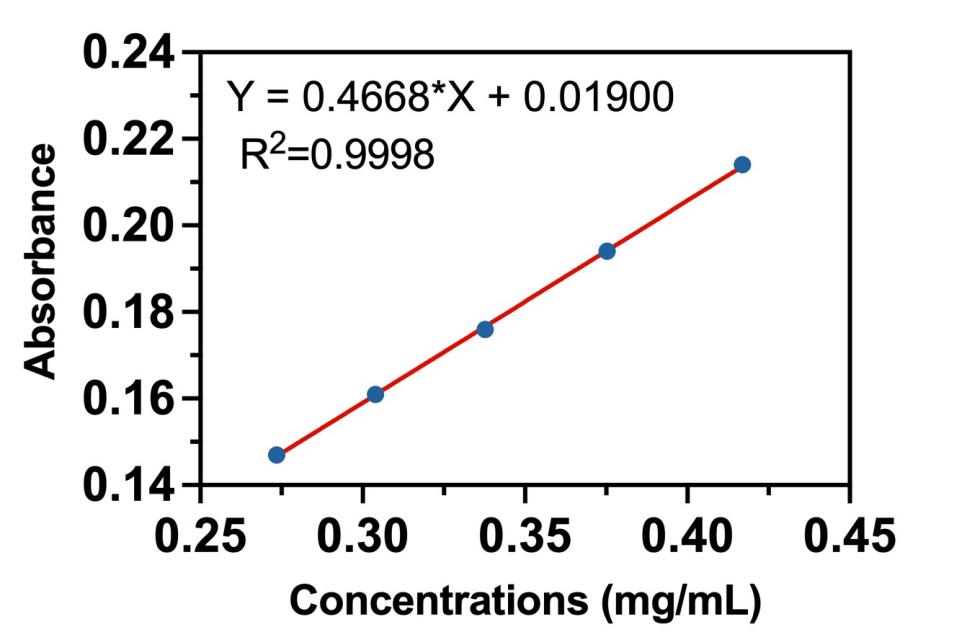


Figure S3. The absorbance UV-Vis spectrum of the levobupivacaine in our study.


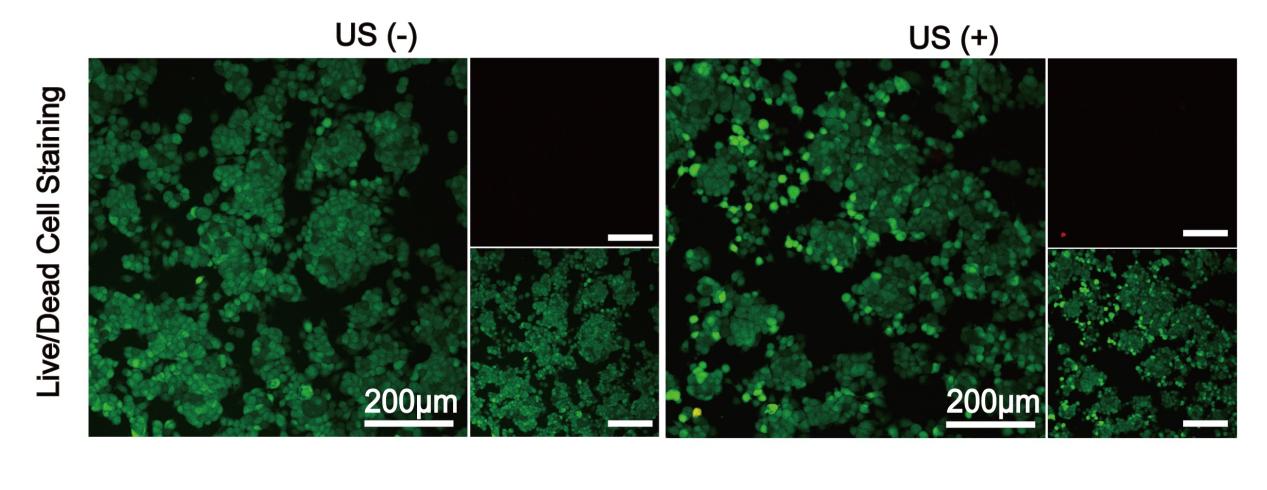


Figure S4. Calcein-AM and PI co-staining images of the RLBP nanoparticles with or without ultrasound irradiation.

Figure S5. Generation of ROS by DCFH staining of DRG cells after being treated with RLBP and RLBP+US. The scale bars are 50 μm.

Figure S6. Representative H&E images of sciatic nerve and muscle at (A) 3 days and (B) 28 days after levobupivacaine + US and the RLBP + US treatments.


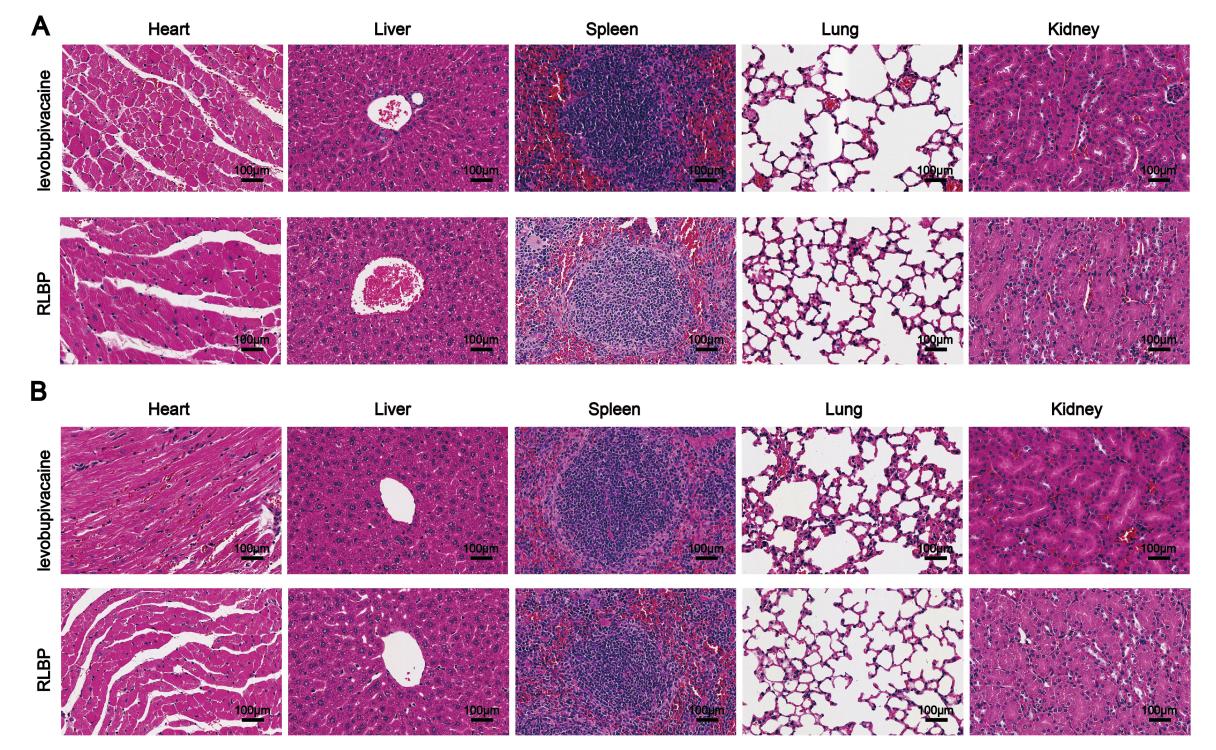


Figure S7. H&E staining sections of the major organs from the mice at (A) 3 days and (B) 28 days after treatment by different pain management strategies. The scale bar is 100 μm.
